# Effect of climate change on phytochemical diversity, total phenolic content and in vitro antioxidant activity of *Aloe vera* (L.) Burm.f.

**DOI:** 10.1186/s13104-017-2385-3

**Published:** 2017-01-25

**Authors:** Sandeep Kumar, Amita Yadav, Manila Yadav, Jaya Parkash Yadav

**Affiliations:** Department of Genetics, M.D. University, Rohtak, Haryana 124001 India

**Keywords:** Antioxidant, *Aloe vera*, Agro-climatic zones, Phytochemicals, Methanol, Total phenolic content (TPC)

## Abstract

**Background:**

The aim of the present study was to analyse the effect of climate change on phytochemicals, total phenolic content (TPC) and antioxidant potential of methanolic extracts of *Aloe vera* collected from different climatic zones of the India.

**Methods:**

Crude methanolic extracts of *A. vera* from the different states of India were screened for presence of various phytochemicals, total phenolic content and in vitro antioxidant activity. Total phenolic content was tested by Folin–Ciocalteau reagent based assay whilst DPPH free radical scavenging assay, metal chelating assay, hydrogen peroxide scavenging assay, reducing power assay and β carotene-linoleic assay were used to assess the antioxidant potential of *A. vera* methanolic leaf extracts.

**Results:**

Alkaloids, phenols, flavonoids, saponins, and terpenes were the main phytochemicals presents in all accessions. A significant positive correlation was found between TPC and antioxidant activity of different accessions. Extracts of highland and semi-arid zones possessed maximum antioxidant potential. Accessions from tropical zones showed the least antioxidant activity in all assays.

**Conclusions:**

It could be concluded that different agro-climatic conditions have effects on the phytochemicals, total phenolic content (TPC) and antioxidant potential of the *A. vera* plant. The results reveal that *A. vera* can be a potential source of novel natural antioxidant compounds.

## Background

Climate change is causing noticeable effects on the life cycle, distribution and phyto-chemical composition of the world’s vegetation, including medicinal and aromatic plants. The changing temperatures and wind patterns associated with climate change are affecting precipitation and thereby plant architecture, flowering, fruiting, phyto-chemical composition and in situ competition with other species. India’s climate is largely controlled by an annual monsoon, appears to be experiencing increasingly severe and erratic precipitation. Therefore, there is a need to understand the effect of higher temperature, various precipitation levels and different soil moisture and fertility, by growing them under such climates and determine how variation in temperature, moisture and edaphic factors might affect the plants’ phenology, nutrient, antioxidant and secondary metabolites levels.

Antioxidative action is one of the prime physiological functions that protect living organisms from oxidative damage caused by reactive oxygen species (ROS). Reactive oxygen species is a term used to describe a number of reactive molecules and free radicals derived from molecular oxygen. During daily activities and with advanced age, oxidative substances and free radicals accumulate in cells affecting various organs and systems in our body. Reactive oxygen and nitrogen species are free radicals that are common outcome of normal aerobic cellular metabolic processes [1, 2]. Unbarred generation of these free radicals leads to attack on various biomolecules, cellular machinery, cell membrane, lipids, proteins, enzymes and DNA causing oxidative stress and ultimately cell death [3]. Overproduction of free radicals can lead to many chronic diseases such as rheumatoid arthritis, atherosclerosis, cancer, diabetes, post-ischemic perfusion injury, cardiovascular diseases, myocardial infarction, chronic inflammation, stroke and septic shock, aging and other degenerative diseases in humans [4, 5]. Human body has an intrinsic system of various enzymes and nutrients to protect from these free radicals. Some extrinsic nutrients are required by many people who are subjected to higher production of free radicals due to stress or physical activities. Micronutrients like vitamin E, β carotene, and vitamin C are the major antioxidants which could be provided in the daily diet as our body cannot produce these nutrients [6]. Protection against free radicals can also be enhanced by taking sufficient amounts of exogenous antioxidants [7].

Antioxidants are used to counterbalance the effect of free radicals. An antioxidant is a stable molecule which donates an electron to a charged free radical and terminates the chain reaction before vital molecules are damaged. Free radical scavenging property of antioxidants delays or inhibits cellular damage [8]. Antioxidants are in great demand as they help in reducing ageing signs. Previously many plants have been screened for their antioxidant potential. Thus, there is growing interest in replacing synthetic antioxidants because of the concern over the possible carcinogenic effects of these in foods with natural ingredients [9]. Primary sources of naturally occurring antioxidants for humans are fruits, vegetables and spices. Medicinal plants contain a wide variety of free radical scavenging molecules such as phenolic compounds (phenolic acids, flavonoids, catechins, proanthocyanidins, quinones, coumarins, tannins etc.), nitrogen compounds (alkaloids, amines, betalains etc.), vitamins, terpenoids, carotenoids and other secondary metabolites which are reported to have antioxidant activity [10–13]. Efforts have been made to search for novel natural antioxidants from tea, fruits, vegetables, herbs, and spices. Herbs are on focus of whole world as a source of novel antioxidants compounds due to their safety as compared to synthetic antioxidants.

*Aloe vera* (syn.: *Aloe barbadensis* Miller) belongs to the Xanthorrhoeaceae family and is a perennial succulent plant [14]. Out of more than 400 species, only a few species of *Aloe* have been considered for commercial importance. *A. vera* being the most potent and, thereby, the most popular plant in the research as well as marketing field [15]. *A. vera* is a rich source of over 200 naturally occurring nutrients which are water soluble and fat soluble vitamins, minerals, enzymes, polysaccharides, phenolic compounds and organic acids [16]. Its secondary metabolites have multiple properties such as anti-inflammatory, antibacterial, antioxidant, immune boosting, anticancer, anti-ageing, sunburn relief and antidiabetic potentials [17–19]. Several traditional uses also have been reported such as for burn injury, eczema, cosmetics, inflammation, fever etc. [20]. This present study focuses on comparative phytochemical analysis, total phenolic content and antioxidant potential of methanolic extracts of *A. vera* from 6 different agro-climatic zones of India. India is a home to an extraordinary variety of climatic regions, ranging from tropical in the south to temperate and alpine in the Himalayan north, where elevated regions receive sustained winter snowfall. These vast climatic variations may cause a difference in the phytoconstituents of the plant species. This study is the first attempt to evaluate antioxidant potential of different *A. vera* accessions from different agro-climatic zones of India.

## Methods

### Collection of plant material

**Fig. 1:**
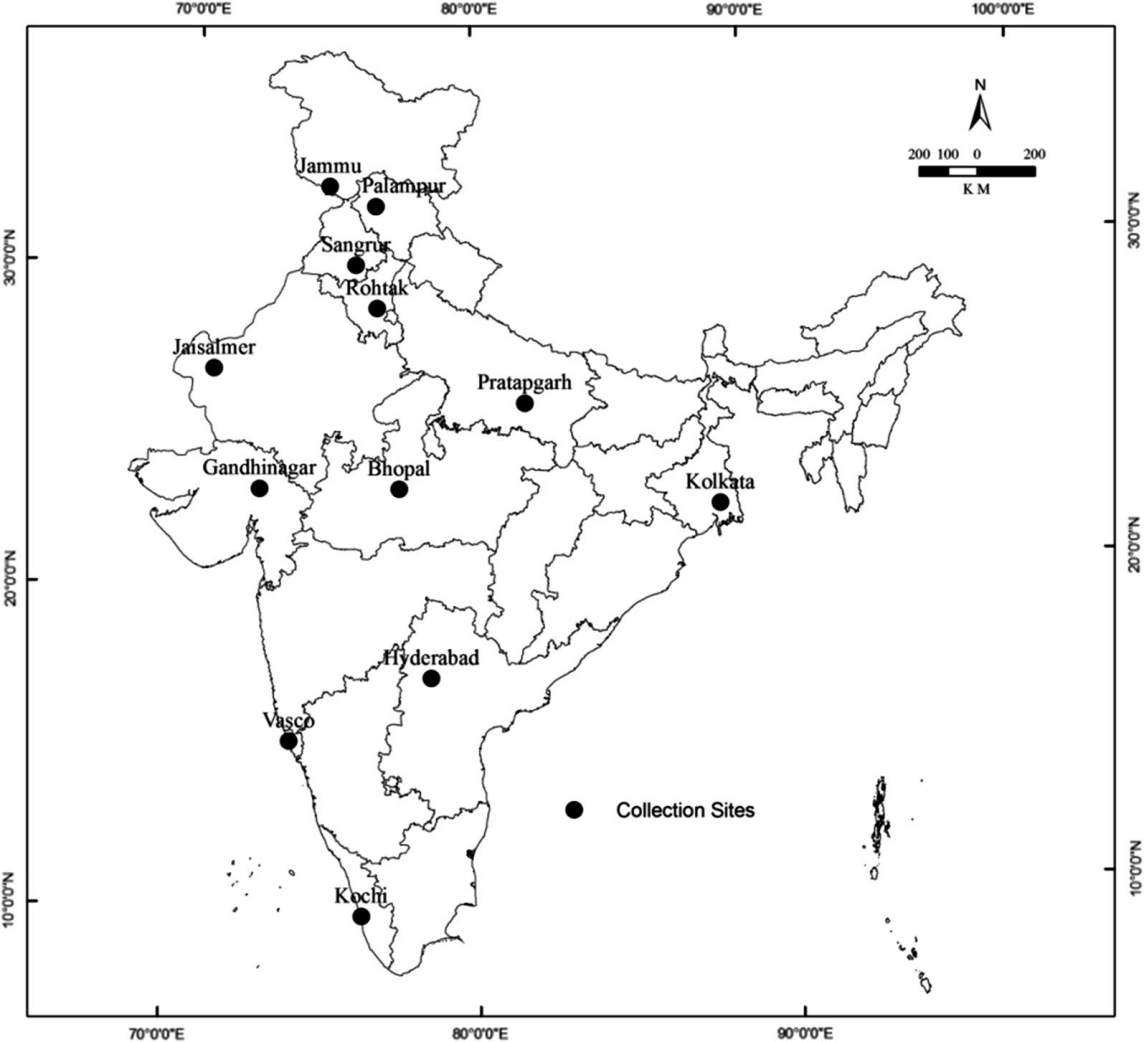
Showing different collection sites from 6 agro-climatic zones of India

Samples were collected from 12 different sites covering 6 agro-climatic zones of India. Each zone had 2 sites (Fig. 1). Collection sites geographical locations along with their average temperature and rainfall are depicted in Table 1. Samples were collected in the months of Jan–Feb 2013. Healthy leaves of *A. vera* were collected from individual plants at each location. The plant material was identified and authenticated by comparing the herbarium specimen (MDU-6803) available in Department of Genetics, M. D. University, Rohtak (India). Samples were placed in sterile plastic bags and were brought to the laboratory in an ice box for analyses.

**Table 1:** Showing plant collection sites along with their average temperature and rainfall

Agro-climatic zones	Accessions	Place of collection	Latitude	Longitude	Average temp. (°C).	Average rainfall (mm).
Highland	Jammu and Kashmir (J&K)	Jammu	32°72′N	74°85′E	7–20	1011
	Himachal Pradesh (H.P.)	Palampur	32°11′N	76°53′E	10–17	1251
Semi-arid	Punjab	Sangrur	30°24′N	75°84′E	15–35	649
	Haryana	Rohtak	28°89′N	76°60′E	20–34	617
Arid	Rajasthan	Jaisalmer	25°55′N	70°57′E	22–35	209.5
	Gujarat	Gandhinagar	23°21′N	72°63′E	22–33	1107
Humid Subtropical	Uttar Pradesh (U.P.)	Pratapgarh	25°89′N	81°94′E	19–32	904
	Madhya Pradesh (MP)	Bhopal	23°25′N	77°41′E	19–32	1146
Tropical wet and dry	West Bengal (W.B.)	Kolkata	22°34′N	88°24′E	22–32	1582
	Telangana	Hyderabad	17°20′N	78°30′E	23–30	812.5
Tropical wet	Kerala	Kochi	09°93′N	76°26′E	24–32	3005
	Goa	Vasco	15°24′N	73°50′E	23–32	3055

### Chemicals

Chemicals and reagents used are Gallic acid, Folin- Ciocalteu reagent, 2,2 diphenyl-2-picrylhydrazyl (DPPH), Trichloroacetic acid (TCA), Hydrogen peroxide, Methanol, FeCl_3_, FeCl_2_, Phosphate buffer, Ferrozine, Potassium ferricyanide, β carotene, linoleic acid, Tween 80, Chloroform of Sigma-Aldrich (USA) and Himedia Laboratories Pvt. Ltd. India. All solvents used were of analytical grade.

### Preparation of extracts

The leaves of the selected plants were collected and washed under running tap water to remove dust and dirt. The washed leaves were cut into small pieces and air dried for few days. Dried leaves were crushed into coarse powder with wiley mill and stored in polythene bags for further use. The methanolic extracts were prepared by cold percolation method. The solution and residue were separated by a centrifuge at a rolling speed of 4000 rpm. A reddish brown colloid containing *Aloe* extract was obtained. Chloroform and sulphuric acid were used to remove the chloroform extractive [21]. After the chloroform evaporation, a yellowish-brown colloid was obtained as crude extract. Stock solution was prepared by dissolving in methanol.

### Phytochemical screening of *A. vera* extracts

Phytochemical constituents of *A. vera* can be classified into nine categories: anthraquinones, inorganic compounds, essential and non essential amino acids, fatty acids, alkaloids, carbohydrates, enzymes, vitamins, and other miscellaneous compounds [22]. *A. vera* methanolic extracts were subjected to phytochemical screening for the presence or absence of various phytochemicals. Phytochemicals were tested in methanolic extracts according to standard protocols [23, 24].

### Determination of total phenolic content

The total phenolic content of the obtained extracts was spectrometrically analyzed by Folin–Ciocalteu method [25]. Gallic acid was used as standard for TPC estimation [26]. Final volume of 5 ml was made by adding 500 µl of Folin–Ciocalteau reagent, 1.5 ml of 20% Na_2_CO_3_ and 2 ml of distilled water to 1 ml (1 mg/ml) of each extract. The mixture was incubated at room temperature for 30–40 min and the absorbance of the developed colour was recorded at 765 nm using UV–Vis spectrophotometer. Calibration curve was constituted using various concentrations of gallic acid ranging from 20 to 100 µg/ml. Total phenolic value was obtained from the regression equation: y = 0.056x + 1.454 with R^2^ = 0.9967 and expressed as mg/g gallic acid equivalent using the formula, C = cV/M; where C = total content of phenolic compounds in mg/g GAE, c = the concentration of gallic acid established from the calibration curve, V = volume of extract and M = the weight of the extract.

### Antioxidant assays

DPPH free radical scavenging assay, hydrogen peroxide scavenging assay, reducing power assay, metal chelating assay and β carotene-linoleic assay were used to assess the antioxidant potential of *Aloe vera* methanolic leaf extracts. Each experiment was done in triplicates and mean values were interpreted to conclude the results.

### DPPH [(1, 1-diphenyl-2-picrylhydrazyl)] free radical scavenging activity

Antioxidant activity of *A. vera* extracts were measured in terms of hydrogen donating radical scavenging ability using the stable DPPH method of Zhu et al. [27]. The reaction was monitored as a color change from purple to pale yellow. A quantity of 1 ml (1 mg/ml) of *Aloe vera* extract was added to 2 ml of 0.5 mM DPPH solution in methanol and the reaction mixture was kept in the dark for 45 min. Absorbance was recorded at 517 nm against blank using a UV–Vis spectrophotometer. Ascorbic acid was used as a standard. The radical scavenging activity on DPPH was expressed as,

$${\text{Scavenging activity }}(\%) = [({\text{A}}_{0} - {\text{A}}_{1} / {\text{A}}_{0} ] \times 100$$

where A_0_ is the absorbance of control and A_1_ is the absorbance of sample extract or standard.

### Hydrogen peroxide scavenging (H_2_O_2_) assay

The ability of extracts to scavenge hydrogen peroxide was estimated by following the method of Ruch et al. [28]. The extract was dissolved in a phosphate buffer at a concentration of 1 mg/ml. A quantity of 1 ml of extract was added to 3.4 ml of phosphate buffer (50 mM, pH 7.4) followed by 600 µl of 400 mM H_2_O_2_. The solution was kept at room temperature for 40 min and absorption was measured at 230 nm. Ascorbic acid was used as control.

The percentage of hydrogen peroxide scavenging was calculated as follows:

$$ {\text{Scavenged }} {\text{H}_{2} \text{O}_{2}} \, (\%) = [\text{A}_\text{i} - \text{A}_\text{t}] / \text{A}_\text{i} \times 100$$

where A_i_ was the absorbance of control and A_t_ was the absorbance of test samples.

### Reducing power assay

The reductive potential of the extract was determined according to the method of Oyaizu [29]. Extracts and standard ascorbic acid (1 mg/ml) were mixed with 2.5 ml of phosphate buffer (0.2 M, pH 6.6) and 2.5 ml potassium ferricyanide (1% w/v). A quantity of 2.5 ml of chloroacetic acid (10% w/v) was added and the solution was centrifuged for 10 min at 3000 rpm. The upper layer solution of 2.5 ml was separated and mixed with 2.5 ml of distilled water and 0.5 ml of FeCl_3_ (0.1% w/v). Absorbance was measured at 700 nm. The increase in absorbance of the reaction mixture indicated reducing power of different *A. vera* accessions.

### Metal chelating activity

The ability of the extract to chelate iron (II) was estimated based on the method of Dinis et al. with minimal modifications [30]. A quantity of 2 ml of extract at a concentration of 1 mg/ml was added with 0.25 ml of 250 mM FeCl_2_. The reaction was initiated by the addition of 0.25 ml of ferrozine. The mixture was shaken vigorously and left at room temperature for 10 min. Absorbance of the solution was measured spectrophotometrically at 562 nm. Methanol was used as blank and a control without extract was also included. The percentage of inhibition of Ferrozine-Fe^2+^ complex formation was calculated from:

$${\text{Inhibition }} (\%) = [({\text{A}}_{0} - {\text{A}}_{1} / {\text{A}}_{0}] \times{{100}}$$

where A_0_ is the absorbance of control and A_1_ is the absorbance of sample extract or standard.

### Antioxidant activity in linoleic acid model system

The antioxidant activity of the extract was assayed based on the β carotene bleaching method developed by Velioglu et al. [12]. An amount of 1 mg β carotene was dissolved in 1 ml of chloroform, and was added with 40 mg of linoleic acid and 400 mg of Tween 80. Chloroform was evaporated with the help of rotary evaporator. Semisolid residue, left after chloroform evaporation was added with 100 ml distilled water with vigorous shaking. A quantity of 0.2 ml of the extract (1 mg/ml) was then added to 5 ml of the prepared solution. Absorbance was measured at zero time and after 60 min incubation at 50 °C. Solution without β carotene was used as blank. In β carotene-linoleic acid assay, the antioxidant capacity was determined by measuring the inhibition of the volatile organic compounds and the conjugated diene hydroperoxides arising from linoleic acid oxidation [31].

$${\text{Antioxidant activity}} = [1 - {\text{ (A}}_{{\text{0}}} - {\text{ A}}_{{\text{t}}} {\text{)/(A}}_{0}^{'} - {\text{A}}_{{\text{t}}}^{'} )] \times 100$$

where A_0_ and A′_0_ are the absorbance at 0 min of the incubation for test sample and control, respectively, and A_t_ and A′_t_ are the absorbance after incubation for 60 min for test sample and control, respectively.

### Statistical analysis

All experiments were performed in triplicates. All values are expressed as mean ± standard deviation (SD) of three separate experiments using the computer programme Excel. Linear correlation between total phenolic content and different antioxidant assays were calculated to establish a relationship between phenol content and its antioxidant potential from different extracts. Correlation was calculated with the help of MS excel programme and SPSS 16.0 version.

## Results

### Phytochemical screening

A wide range of various phytochemicals; alkaloids, glycosides, reducing sugars, phenolic compounds, steroids and terpenoids, flavonoids, tannins and saponin glycosides were tested with their appropriate protocols and reagents. The *A. vera* methanolic extracts showed presence of most of the phytochemicals tested; though amounts varied in the different accessions. The comparative presence of phytochemicals in the different crude extracts is depicted in Table 2.

**Table 2:** Qualitative analyses of the phytochemical components of *Aloe vera* extracts

Phytochemical analysis			Collection sites											
Chemical constituents	Test reagent	Observation	1.	2.	3.	4.	5.	6.	7.	8.	9.	10.	11.	12.
Alkaloids	Mayer’s reagent	White ppt	+++	+++	+++	+++	++	++	++	++	++	++	+	++
Glycosides	10% Lead acetate solution	White ppt	+++	++	+++	++	++	+++	++	+++	+++	++	++	++
Reducing sugar	Benedict’s solution	Reddish brown ppt	++	++	++	+++	++	++	+++	++	++	+++	++	+++
Phenolic compounds	Ferric chloride solution	Green colour	+++	+++	+++	+++	++	++	++	++	++	++	++	++
Steroids and terpenoids	Acetic anhydride and conc: H_2_SO_4_	Pink colour	++	++	+	++	++	+	+	+	++	+	+++	++
Flavonoids	Benzene FeCl_3_	Yellow ppt	+++	+++	+++	+++	++	++	+++	++	++	++	++	++
Tannins	FeCl_3_ and 10% lead acetate	White ppt	++	++	++	++	++	+	++	++	++	+	++	+
Saponin glycosides	Distilled water	Frothing take place	+++	+++	++	++	++	++	++	++	++	++	++	++

***+++*** high, **++** medium, **+** low

### Determination of total phenolics

The phenolic compounds are oxidized to phenolates by the reagent at alkaline pH in a saturated solution of sodium carbonate resulting in a blue complex. All accessions showed the presence of total phenols in crude extracts. Table 3 enlists the different TPC values obtained for all 12 accessions. The values ranged from 32.9 to 65.7 mg GAE per g of dry weight. Maximum values of TPC were obtained for Punjab, Jammu and Himachal accessions. Kerala, Telangana and West Bengal showed low TPC values as compared with other accessions.

**Table 3:** Total phenolic contents of *Aloe vera* extracts

Accession name	Total phenolic content (mg of GAE/g of extract)
Jammu	63.2 ± 0.15
Himachal Pradesh	62.3 ± 0.26
Haryana	58.4 ± 0.35
Panjab	65.7 ± 0.30
Rajasthan	56.9 ± 0.23
Gujarat	54.6 ± 0.41
Uttar Pradesh	55.1 ± 0.15
Madhya Pradesh	52.5 ± 0.28
West Bangal	46.4 ± 0.76
Telangana	38.9 ± 0.35
Goa	53.4 ± 0.24
Kerala	32.9 ± 0.19

### Antioxidant activity

The 12 crude extracts of *A. vera* were investigated for their antioxidant potential by using different methods and all assays showed the antioxidative potential of the extracts. All extracts showed significant antioxidant activity ranging from 56 to 80% with different climatic conditions showing significant differences on the antioxidant potential of the plant species.

### DPPH free radical scavenging activity

**Fig. 2:**
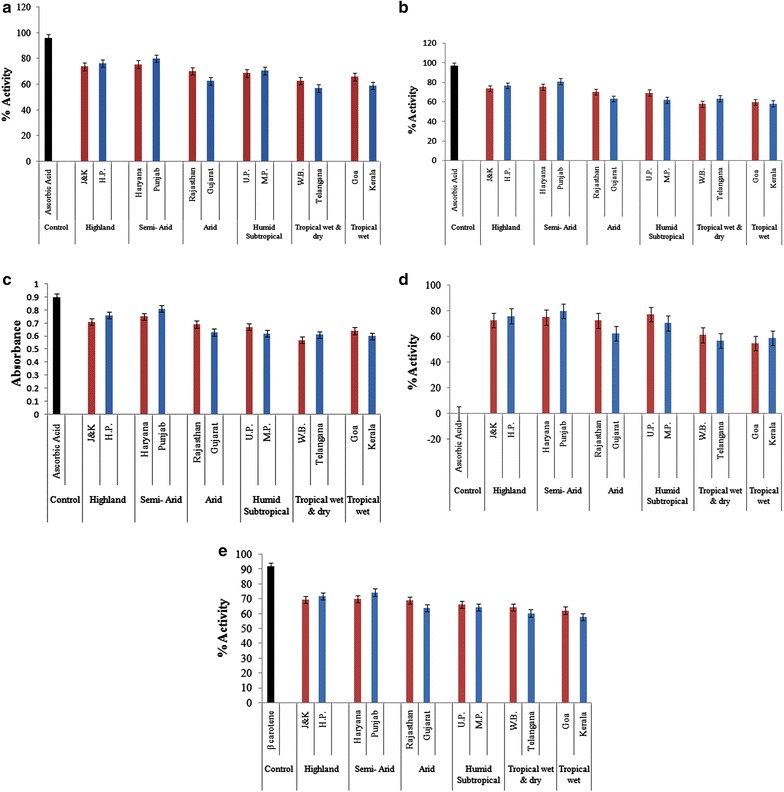
Antioxidant activity of *Aloe vera* accessions with control by using different assays. **a** Showing DPPH free radical scavenging activity of *Aloe vera* and ascorbic acid. **b** Showing hydrogen peroxide scavenging activity of *Aloe vera* and ascorbic acid. **c** Showing reducing power activity of *Aloe vera* and ascorbic acid. **d** Showing metal chelating activity of *Aloe vera* and ascorbic acid. **e** Showing reductive potential of *Aloe vera* leaf extracts assayed by β carotene-lenoleic acid assay

The reduction capability of DPPH radical was determined by the decrease in absorbance at 517 nm induced by the antioxidants. The highest antioxidant capacity observed was 80%. Punjab, Himachal Pradesh and Haryana showed a high antioxidant capacities ranging from 75.54 to 80.2%. Telangana, Gujarat and West Bengal showed least antioxidant capacity ranging from 56.75 to 62.66%. Figure 2a shows the percentages of free radical scavenging activity of different aloe accessions. Ascorbic acid showed 96% radical scavenging activity.

### Hydrogen peroxide scavenging (H_2_O_2_) assay

Reduction potential of methanolic extracts of *A. vera* in H_2_O_2_ assay ranged from 58.54 to 81.10%. Reducing activity of ascorbic acid was 97%. Punjab, Telangana and Himachal Pradesh accessions showed maximum activity. These were followed by Haryana, Rajasthan and West Bengal accessions (Fig. 2b).

### Reducing power assay

The data observed on the reducing power of the extracts contribute significantly towards the antioxidant effects. Punjab, Himachal Pradesh and Haryana had high absorbance values that indicate their greater reductive potential and electron donor ability for stabilizing free radicals. Other accessions also showed almost similar observations to H_2_O_2_ assay. Figure 2c represents activity of all extracts and ascorbic acid with respect to their absorbance values.

### Metal chelating activity

In the presence of other chelating agents, the complex formation was disrupted with the result that the red colour of the complex was decreased. Ferrous ions are also commonly found in food systems and considered as prooxidants. Ferrozine form complexes with the ferrous ion, generating a violet color [32]. Punjab, Himachal Pradesh and Uttar Pradesh accessions showed high metal chelating activity. Kerala, Telangana and Goa showed least activity. Control ascorbic acid showed no activity. Activity of all extracts with respect to their absorbance values are represented in Fig. 2d.

### Antioxidant activity in linoleic acid model system

Total antioxidant activities of the methanolic extracts of *A. vera* were determined using β carotene- linoleic acid model system. Figure 2e shows the reductive potential of *A. vera* leaf extracts assayed by β carotene-lenoleic acid assay. All extracts showed reductive potential. Antioxidant potential ranged from 59.60 to 74.4%. Maximum activity was observed for Punjab, Jammu, Haryana and Himachal Pradesh accessions and minimum activity was for Goa, Telangana and Kerala.

### Correlation analysis

**Fig. 3:**
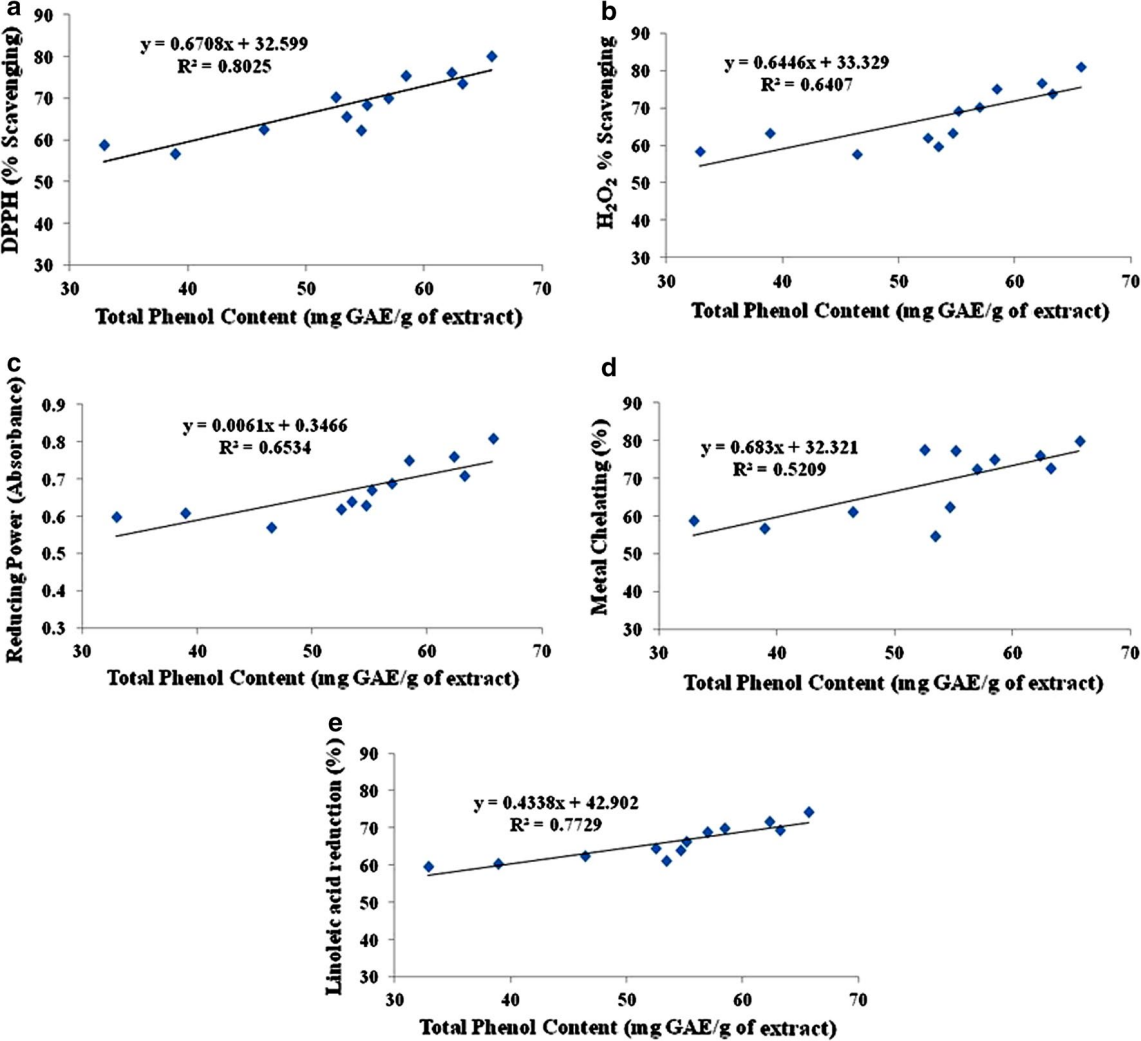
Linear correlation between total phenolic content and different antioxidant assays. **a** Showing linear correlation between total phenolic content and DPPH free radical scavenging activity. **b** Showing linear correlation between total phenolic content and hydrogen peroxide scavenging activity. **c** Showing linear correlation between total phenolic content and reducing power activity. **d** Showing linear correlation between total phenolic content and metal chelating activity. **e** Showing linear correlation between total phenolic content and β carotene linoleic acid assay

Satisfactory correlations have been reported reported between the parameters of antioxidant activity and the content of total phenols in different in vitro models, with different extracts of plant. Figure 3a–e illustrates the linear correlation between total phenols contents and antioxidant activity of different extracts. R^2^ values were 0.8025, 0.6407, 0.6534, 0.5209, 0.7729 for DPPH free radical scavenging assay, Hydrogen peroxide scavenging assay, Reducing power assay, metal chelating assay and β carotene-linoleic assay respectively. The matrices of linear correlation between total phenolic content and different antioxidant assays were analyzed (Table 4). They also showed good correlation with respect to each others.

**Table 4:** Linear correlation between total phenolic content and different antioxidant assays by using Pearson-correlations method

	Phenol	DPPH	H_2_O_2_	Reducing power	Metal chelating	Linoleic reduction
Phenol	1.000					
DPPH	0.895^a^	1.000				
H_2_O_2_	0.800^a^	0.868^a^	1.000			
Reducing power	0.808^a^	0.900^a^	0.970^a^	1.000		
Metal chelating	0.721^a^	0.853^a^	0.772^a^	0.719^a^	1.000	
Linoleic reduction	0.879^a^	0.938^a^	0.952^a^	0.935^a^	0.846^a^	1.000

^a^Correlation is significant at the 0.01 level (2-tailed)

## Discussion

There are numbers of secondary metabolites found in plants which contribute significant biological activities [33]. Plant materials contain numerous types of antioxidants with varied activities [34, 35].

*Aloe vera* is a perennial succulent xerophyte originated in South Africa, although it is native to dry subtropical and tropical regions [36]. The plant species is are drought resistant and able to tolerate a wide range of climatic situations. Hot humid conditions along with high rainfall are the most suitable [37].

Phytochemical analysis of plants is commercially important being great interest of pharmaceutical industries for the production of new drugs to cure various diseases [38]. Our study showed the presence of various medicinally important phytoconstituents from the different *A. vera* accessions in good amount, and hence rationalizes the use of this plant species as an herbal remedy. Extracts having high phenolic content also showed good antioxidant activity. Linear correlation analyzed between the total phenolic content and the different antioxidant assays by using Pearson-correlations method showed the significant correlation at 0.01 levels (2-tailed). Previous studies also have concluded that there was a significant linear correlation between total phenolic content and antioxidant potential [39, 40].

Our study emphasizes the antioxidant potential of *Aloe vera* methanolic leaf extracts. Previous studies on *A. vera* stated that the plant contains substantial amounts of antioxidants including α-tocopherol (vitamin E), carotenoids, ascorbic acid (vitamin C), flavonoids, and tannins [41]. Glutathione peroxidase activity, superoxide dismutase enzymes and a phenolic antioxidant were found to be present in *A. vera* gel, which may be responsible for these antioxidant effects [42]. Methanolic extract induced the best extraction yield and more complex composition of phenolics [43]. The methanol extracts of *A. vera* were also screened previously for their in vitro antioxidant activity, and extracts exhibited good antioxidant activity [44]. Potent antioxidative compounds like Aloe barbendol, Aloe emodin, barbaloin A and Aloe chrysone have been isolated from methanolic extracts of *A. vera*. [45]. Biological activities of *A. vera* may be due to the synergistic action of these compounds, rather than from a single defined component [46].

Five different methods were employed for determining antioxidant potential of *Aloe vera* leaf extracts. DPPH radical scavenging method is an extensive procedure to evaluate the free radical scavenging ability of various samples [47]. The effect of antioxidants on DPPH radical scavenging was supposed to be due to their hydrogen- donating ability. Hydrogen peroxide is generally not very reactive, but it can sometimes be toxic to cell because it can give rise to hydroxyl radicals in the cells. Thus, the removal of H_2_O_2_ is essential for antioxidant defence in cell or food systems. In general, the reducing properties are associated with the presence of reductones, which have been shown to exert antioxidant action through breaking the free radical chain by donating a hydrogen atom [48, 49]. In the reducing power assay, the presence of reductants in the sample would result in the reduction of Fe^3+^ to Fe^2+^ by donating an electron [50]. Metal chelating activity is based on chelation of Fe^2+^ ions in a quantitative manner by the reagent ferrozine, which results to the formation of a complex with Fe^2+^ ions [51]. So the chelating ability influences other scavenging activities of free radicals which protect the organisms against oxidative damage. Mechanism of β carotene bleaching is a free radical mediated phenomenon resulting from the formation of hydroperoxides from linoleic acid oxidation. The presence of antioxidant extracts can hinder the extent of β carotene bleaching by acting on the linoleate- free radical and other formations of free radicals in the system [52, 53].

India is characterized by strong temperature and rainfall variations along with other different environmental and climatic fluctuations in different seasons [54]. Phytochemical composition of plants is greatly influenced by different agro-climatic conditions. According to previous findings it was suggested that environmental temperature plays a significant role on antioxidant activity evaluation and it is more pronounced in cold weather [55]. Therefore, we collected the plant material in the winter season (Jan–Feb 2013). In the present study we also found that the extracts of highland and semi-arid zones possess maximum antioxidant potential. Accessions from Tropical zones showed least antioxidant activity in all assays. *A. vera* can grow in almost all types of environmental conditions but there are several factors that can affect the quality and quantity of a particular constituent. Previous studies state that phytochemical composition of plants is influenced by a variety of environmental factors including the geography, climate, soil type, sun exposure, grazing stress, seasonal changes etc. [56–58].

*Aloe vera* is a cold sensitive plant. During stress more phytochemicals are produced in plants to withstand the adverse conditions. Studies conducted on plants in stress conditions showed higher production of flavonoids, anthocyanins and mucilaginous substances [59]. Our previous finding on antimicrobial activity and quantification of aloe- emodin on the same *A. vera* extracts also supports the present statement [60].

Report by Sanders [61], describing that an increase in unsaturated fatty acids is generally associated with cooler climates leading to production of antioxidants for a self- defense system against environmental stress, supports our present findings to suggest pronounced effects of environmental temperature on the different *A. vera* extracts. Lower temperature leads to higher production of phenolics and vice versa [62]. Plants from hilly areas are thought to be more important from nutritional and dietary points of view [63]. *A. vera* is found to grow in hot humid and high rainfall conditions, so semi- arid conditions having low rainfall are also not favorable for the plant. Although, soil texture and climatic conditions of semi-arid region is normally most suitable for majority of plant crops, *Aloe* genus however is an exception. In present study, accessions from colder regions showed the good antioxidant activity, supporting the statement that more phytochemicals are produced in plants under stress [64]. Consequently, phytochemical content can vary with growing environmental conditions. From the present work, it could be concluded that agro- climatic locations along with temperature and rainfall have significant effects on the *A. vera* plant phytoconstituents and its antioxidant potential. However, there is still a need to investigate the effects of soil properties and other related biotic and abiotic factors.

## Conclusion

The present study showed that *A. vera* is a promising source of bioactive phenol which has a good antioxidant activity. The study demonstrated that the total phenolic content and antioxidant activity were higher in *A. vera* plants grown in Northern India in comparison to Southern India. Phytochemical analysis in relation to their activity from different climatic regions can help in selecting places for mass production of this plant species to enhance its pharmaceutical and marketing values. The present work tries to establish a correlation between phytochemicals, TPC and antioxidant activity of *A. vera* from different agro-climatic zones of India and have found that climatic zones showed significant effect on all these parameters. Our study emphasizes that north Indian climatic conditions are more suitable for the antioxidant potential of *A. vera*. Good antioxidant properties of the Aloe could be considered for applications in food, medicine and cosmetic industries.
